# Electrocortical changes associating sedation and respiratory depression by the opioid analgesic fentanyl

**DOI:** 10.1038/s41598-019-50613-2

**Published:** 2019-10-01

**Authors:** Gaspard Montandon, Richard L. Horner

**Affiliations:** 1 Department of Medicine, Faculty of Medicine, University of Toronto, Toronto, Canada; 2 Keenan Research Centre for Biomedical Sciences, Unity Health Toronto - St. Michael’s Hospital, Toronto, Canada; 3 Department of Physiology, Faculty of Medicine, University of Toronto, Toronto, Canada

**Keywords:** Neuroscience, Neurophysiology, Respiration

## Abstract

Opioid drugs are the mainstay of pain management but present the side-effect of respiratory depression that can be lethal with overdose. In addition to their respiratory effect, opioids also induce a profound sedative state and produce electrocortical features characteristic of a state of reduced brain arousal, similar to anaesthesia or sleep. In such states, respiratory activity depends more on the integrity of the brainstem respiratory network than it does during wakefulness. Accordingly, we propose that sedation by fentanyl induces specific electrocortical changes consistent with reduced brain arousal, and that the magnitude of respiratory depression is associated with distinct electrocortical changes. To these aims, we determined the effects of systemic injections of fentanyl (dosage 100 µg ·kg) versus control on electrocortical  and respiratory activities of freely-behaving rats. We found that fentanyl induced electrocortical changes that differed from those observed in sleep or wakefulness. Fentanyl increased δ (1–3 Hz) frequency power (*P* < *0.001*), but reduced α (7.5–13.5 Hz) and β_2_ (20–30 Hz) powers (*P* = *0.012* and *P* < *0.001*, respectively), when compared to wakefulness. Interestingly, respiratory rate depression by fentanyl was significantly correlated with increased θ power (R = 0.61, *P* < *0.001*), therefore showing a clear association between electrocortical activity and the magnitude of respiratory rate depression. Overall, we provide new evidence linking specific electrocortical changes to the severity of respiratory depression by opioids, which highlights the importance of considering the cortical and subcortical effects of opioids in addition to their impacts on breathing when evaluating opioid-induced respiratory depression.

## Introduction

Opioid drugs are widely used to alleviate pain but their effective use is limited by the life-threatening side-effect of respiratory depression^1^. Opioids induce profound respiratory changes, such as sleep apnea and hypoventilation^2^, which can lead to complete respiratory arrest with opioid overdose. Opioid drugs bind to µ-opioid receptors (MOR) in many brain regions and inhibit nociceptive, arousal, and respiratory circuits^3–7^. These drugs depress breathing by acting on key-structures of the brainstem^8^, which include the ventrolateral medulla^9^, the pons^10^, the rostral ventromedial medulla^7^, and the medullary raphe nuclei^11^. Inhibition of these respiratory structures and circuits directly induce respiratory depression by reducing respiratory rate^9^ and respiratory chemosensitivity^11^. In addition to the direct effects of opioid drugs on such respiratory circuits, descending pathways originating from sub-cortical and cortical regions that modulate brainstem circuits are also inhibited by opioid drugs^12^.

In fact, in addition to their analgesic and respiratory properties, opioid drugs also cause a profound sedative state^13^, reminiscent of sedation induced by other medications such as sedatives and anesthetics^14^. This sedative state has been studied in humans using the electroencephalogram (EEG), where infusion of sulfentanyl, a potent µ-opioid receptor ligand, induced a dose-dependent changes in EEG activity. At mild doses, EEG exhibited reduced high-frequency β content and increased of α waves^15^. With increasing doses, the EEG consisted of increased θ and δ slow-wave activity. Similarly, low doses of morphine induced EEG changes consistent with fentanyl, i.e. reduction of β activity^16^. Although distinct from the EEG changes elicited by sleep, the EEG features associated with opioids are characteristic of a state of reduced brain arousal, i.e. reduced β activity and increased low frequency activity. It is unclear, however, how the sedative properties of opioids *per se* may impact on breathing.

In states of reduced brain arousal, such as sleep, respiratory activity depends more on the integrity of the brainstem respiratory network than it does during wakefulness^17,18^. Inhibition of the respiratory network by opioid ligands during states of reduced brain arousal, for instance sleep or anaesthesia, have serious effects on breathing stability and persistence^2,9^. In humans, similarly to sleep-disordered breathing where respiratory events mostly occur during non-rapid-eye-movement (non-REM) and REM sleep, respiratory depression by opioids is more pronounced during sedation^16^. Considering the powerful sedative properties of opioid drugs and their impacts on electrocortical activity, we aim to understand the relationship between changes in arousal and respiratory depression. In addition, mechanistic^19^ and drug discovery studies^20^ rely on rodent models to identify new mechanisms and therapies. A better understanding of both the sedative and respiratory effects of opioids is therefore needed to better assess the potency of new opioid analgesics without the side-effect of respiratory depression. We hypothesized that sedation by fentanyl induces specific electrocortical changes that are consistent with reduced arousal but differ from the electrocortical changes observed with non-REM and REM sleep. We also propose that the magnitude of respiratory rate depression depends on the electrocortical changes induced by opioid drugs, as previously suggested by the state-dependent effects of opioid inhibition of the medulla on breathing^9^.

To test these hypotheses, we determined the effects of fentanyl versus control on electrocortical and respiratory activities in freely-behaving rats. To mimic the impact of a high dosage of fentanyl that occurs during a fentanyl overdose, we used a dosage of 100 µg/kg of fentanyl, which is relatively high in rats^21^. 

We used signal processing methods to identify common behaviours in frequencies between cortical and respiratory activities in response to the opioid analgesic fentanyl. Spectral analyses were applied to the electrocortical signals to identify the frequency regions affected by opioid drugs. We then related these changes to respiratory rate depression to identify the cortical signatures associated with depression by opioids. To detect common behaviors in frequencies between electrocortical and respiratory activities, we used wavelet cross spectrum and associated coherence functions^22^. In a separate set of experiments, we induced anaesthesia in rats and determined whether levels of anaesthesia modified electrocortical activity and how these changes can affect the magnitude of respiratory depression by opioid analgesics.

## Methods

### Animal care and use

All procedures were performed in accordance with the recommendations of the Canadian Council on Animal Care and were approved by the University of Toronto Animal Care Committee. Sixteen adult male Wistar rats weighing between 300–400 g were used for this study (Charles River Laboratories). Eight rats were used for control (saline) injection and eight rats for fentanyl injection. Animals were kept on a standard day-night cycle (lights on at 7 am and off at 7 pm) and all experiments were performed during the day.

### Freely-behaving preparations

The experimental procedures were adapted from a previous study^9^. One week prior to the experiment, sterile surgery was performed under isoflurane anaesthesia (2–3%) to implant the rats with electrocortical (extradural) and postural (neck) muscle electrodes to identify sleep-wake states, and diaphragm electrodes for respiratory muscle recordings. To record diaphragm activity, two wires were sutured onto the costal diaphragm via an abdominal approach. The rat was placed in the prone position in a stereotaxic apparatus (Model SAS-4100) with blunt ear bars, and three holes were drilled into the skull for the placement of the electrocortical electrodes. Holes were drilled without damaging the dura. To measure extradural electrocortical activity, we used stainless steel screws (size 0–80 × 1/16, Plastic One Inc., Roanoake, VA, USA) as electrodes, screwed in skull but positioned above the dura. Two screws were placed approximately 2 mm to the right and 2 mm anterior to bregma, and 2 mm to the left and 3 mm posterior to bregma for electrocortical activity; the third screw was placed 3 mm to the left and 3 mm anterior to bregma. Insulated multi-stranded stainless steel wires were also sutured on the dorsal neck muscles to record the electromyogram. Post-surgical care consisted of a subcutaneous injection of an anti-inflammatory drug (ketoprofen, 2 mg · kg^−1^) and an analgesic (buprenorphine, 1 mg · kg^−1^). The rats recovered for one week prior to experiment.

### Experimental procedures

**Figure 1:**
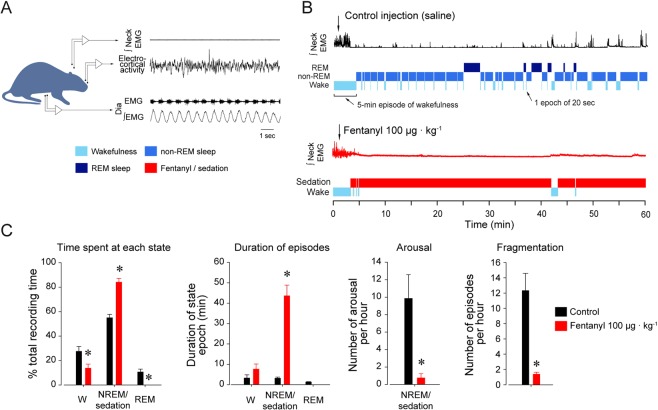
Effects of fentanyl on behaviors and sleep/wake states. Behavioural changes associated with systemic injection of the µ-opioid receptor agonist fentanyl. (**A**) Intraperitoneal injection of saline (control condition) or fentanyl citrate (100 µg · kg^−1^) while recording neck and diaphragm muscle activities, and electrocortical activity in rats. After initially increasing neck muscle activity, presumably as a result of the acute behavioural response to handling and the injection itself, control injection did not further alter sleep architecture and behaviour. (**B**) Systemic fentanyl quickly reduced motor activity and induced a persistent sedative state. (**C**) Fentanyl significantly decreased time spent awake, and increased time spent in a state of sedation, compared to time spent in non-REM sleep in the control condition. The mean duration of episodes of sleep or sedation was significantly increased by fentanyl. Also, arousals occurred significantly less with fentanyl compared to control, and periods of fentanyl-induced sedation were less fragmented by arousal than in control. Dia, diaphragm. EMG, electromyogram. * indicates mean data significantly different than control with *P* < *0.05*. Data are presented as mean ± SEM.

On the day of the experiment, the rat was connected to the recording apparatus through a tether cable which allowed electrophysiological signals to be recorded while the rat moved freely in a plexiglass bowl filled with fresh bedding. The plexiglass bowl was placed on a rotating turntable (Raturn, BASi, West Lafayette, IN, United States) which automatically adjusts its position when the rat moves to avoid entanglements of recording cable. The rat was acclimatized to the recording chamber for about 2 hours. During that time, quality of the signals were checked. A two-hour acclimatization period is usually long enough in rats as they are not overtly stressed or anxious. Rats usually sleep quite well in the chamber after a few hours. Data were amplified, filtered, moving-time averaged (BMA-400 Bioamplifier, CWE Inc) and acquired at a sampling rate of 1,000 Hz using a Micro-1401 data acquisition system and Spike software (Cambridge Electronic Design). Respiratory rate, diaphragm and neck muscle amplitudes were averaged every 10-sec time bin. Electrocortical and neck muscle activities were also displayed every 10 sec to identify the prevailing sleep/wake states according to standard criteria^23^. Averaged baseline values were then recorded over a 30-minute period before fentanyl was injected. To determine the effects of a systemic dose of a µ-opioid receptor acting analgesic on respiratory and electrocortical activities, we administered fentanyl citrate (diluted in saline, 100 µg · kg^−1^, intraperitoneal, Fig. 1A). This dose of fentanyl is comparable to a dose of 4–7 µg · kg^−1^ in humans, and is a relatively high dose in rats^21^. The dose used was however well under neurotoxic levels in rats^24^. We used systemic intraperitoneal injection to avoid severe respiratory depression and respiratory arrest that my lead to brain hypoxemia. After injection, recordings were made for 60 minutes and average values were again calculated for each 10-sec epoch. Data was analyzed and averaged between 30 and 60 min post-injection. In another group of rats, saline (control) was injected under the same conditions to minimize the confounding effects on behaviours due to drug injection and animal handling.

### Signal processing

Using Matlab scripts (Mathworks), the powers of the electrocortical signal were calculated using Fast Fourier Transform and power spectral density as previously described^17^. Briefly, we calculated the power spectral density estimate using the *periodogram* function of Matlab for each 10-sec epoch. We used a 4-second Hamming window which reduced the frequency resolution of our analysis to 0.5 Hz or 2 bins per Hz. Using the powers of these frequency bins, we averaged the power for the following frequency bands: δ (1–4 Hz), θ (4–7.5 Hz), α (7.5–13.5 Hz), β_1_ (13.5–20 Hz), and β_2_ (20–30 Hz), as previously done for sleep studies in rats^9^. These frequency bands are consistent with EEG frequency bands used in polysomnography to quantify sleep-wake states and arousal^25^.

To detect common behaviors in frequencies between electrocortical and respiratory activities, we determined the wavelet cross spectrum and associated coherence function using Daubechies wavelet transform^22^. The application of the cross-spectrum wavelet transform to electrocortical and respiratory activities can reveal localized similarities in time and scale.

### Data analysis

Prevailing sleep/wake states were identified for each epoch according to standard criteria in rats^23^. Following saline injection, each epoch was classified as wakefulness, non-REM, or REM sleep. Wakefulness was characterized by low δ frequencies and high neck muscle activity. Non-REM sleep was characterized by high δ frequencies, low β frequencies, high electrocortical amplitude and low neck muscle activity. REM sleep presented low δ frequencies, high θ frequencies and low neck muscle activity. Because sedation significantly differs from non-REM and REM sleep^14^, we defined epochs with lack of motor activity following systemic injection of fentanyl as epochs of sedation.

### Anesthetized experiments

In anesthetized rats, we injected fentanyl intra-peritoneally while recording the electrocortical and diaphragm muscle activities. The experimental procedures were as described previously^17^. Briefly, we recorded activities of the diaphragm muscle in isoflurane-anesthetized (1–2% inspired), tracheotomized and spontaneously breathing (50% inspired oxygen, balance nitrogen) rats. Diaphragm muscle activity was recorded using stainless steel bipolar electrodes positioned and sutured onto the right crural diaphragm. The electromyogram signal was amplified, band-pass filtered, integrated, and digitized at a sampling rate of 1000 Hz as described for freely-behaving experiments. Rats were kept warm at 36.8 °C with a heating pad during the experiments.

### Statistics

For the freely-behaving experiments, two-way mixed ANOVAs with sleep-wake states being a repeated factor and treatment being the between-subjects factor followed by Holm-Sidak post-hoc tests were used to determine the state-dependent effect of fentanyl versus control on each physiological variable. For comparison between wakefulness, non-REM, REM sleep, and sedation, one-way ANOVAs were used followed by Holm-Sidak post-hoc tests. P < 0.05 was considered significant. For the anesthetized experiments, changes from baseline condition in response to fentanyl at two levels of isoflurane (1% and 2%) were determined with two-way repeated-measure ANOVAs (baseline/fentanyl drug application and isoflurane levels being the repeated factors) followed by Holm-Sidak’s post-hoc tests. P < 0.05 was considered statistically significant. For all figures and text, data are presented as means ± SEM. All statistical tests and figures were prepared with Sigma Plot version 12 (Systat Software Inc.).

## Results

### Behavioral profile associated with sedation

One of the key-features associated with opioid analgesics is sedation. To assess the sedative properties of opioid drugs in rodents, we first compared the behavioral changes associated with systemic saline injection with those induced by systemic fentanyl (100 µg · kg^−1^, Fig. 1B). This comparison is necessary to control for injection and manipulation of the animal. The percentages of time spent in wakefulness, non-REM, and REM sleep following control injection were compared to the percentages of time spent during wakefulness and sedation after fentanyl injection. We defined sedation as episodes of rest after fentanyl injection, i.e. reduced motor activity and reduction in high frequency electrocortical activity. After control injection, animals spent 27.5 ± 3.1% of their time in wakefulness, 55.0 ± 3.0% in non-REM sleep, and 10.7 ± 2.5% in REM sleep (Fig. 1C). In contrast, after fentanyl injection, animals spent 13.8 ± 2.9% of their time in wakefulness, 84.1 ± 3.0% in a sedative state, and 0% time in REM sleep. There were significant differences between control and fentanyl groups in wakefulness, sleep/sedative states, and REM sleep (*P* = *0.003*, *P* < *0.001* and *P* = *0.019* respectively). The durations of wakefulness/sleep episodes, i.e. the average duration of episodes of wakefulness, sleep, or sedation, were also compared (Fig. 1B,C). Animals injected with fentanyl had longer episodes of sedation compared to episodes of non-REM sleep in control animals (*P* = *0.001*, Fig. 1C). The number of arousals per hour was also lower when the animals were sedated (*P* = *0.002*, Fig. 1C), and sedative state was also less fragmented after fentanyl injection compared to non-REM sleep (*P* = *0.025*, Fig. 1C). Overall, these data show that fentanyl induced a persistent sedative state with significant changes in behaviours such as less time in wakefulness, long episodes of sedation, less arousals, and less fragmented episodes of sedation compared to non-REM sleep following control injection.

### Electrocortical signatures of sedation by fentanyl

**Figure 2:**
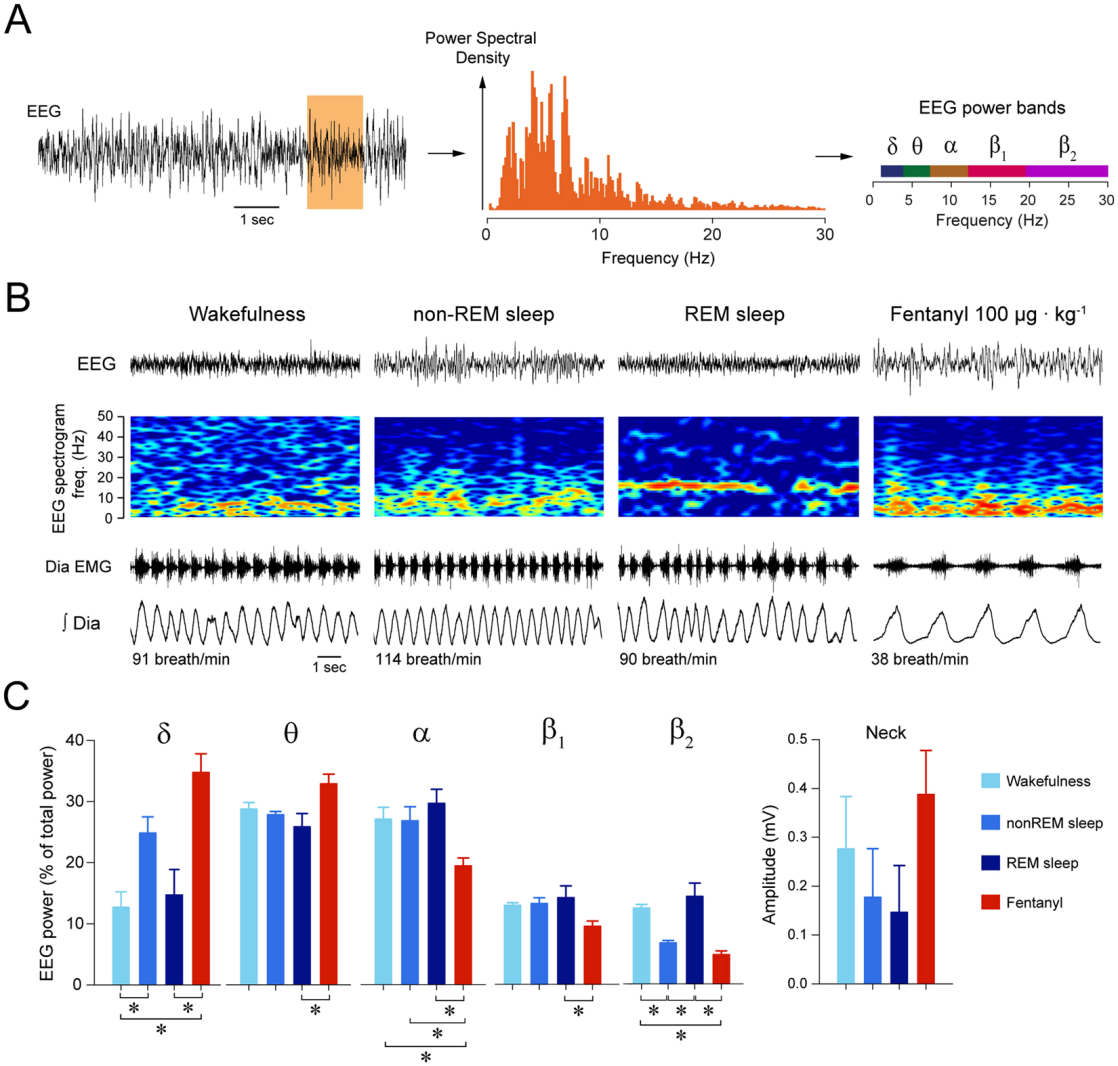
Fentanyl induces distinct electrocortical changes associated with sedation. **(A**) Electrocortical power spectral density and power bands were calculated for each epoch. (**B**) Epochs were then categorized as wakefulness, non-REM sleep, REM sleep, or sedation (following fentanyl 100 µg · kg^−1^). Spectrograms of electrocortical activity, i.e. the distribution of electrocortical powers from 0 to 50 Hz, show that fentanyl increased electrocortical power between 0 and 10 Hz and decreased power between 20–50 Hz compared to wakefulness. (**C**) Mean data showed that fentanyl increased significantly δ power but decreased significantly β_2_ power compared to wakefulness (n = 7). During non-REM sleep, δ power was higher than in wakefulness (n = 7), whereas β_2_ was lower (n = 7). There was no significant difference in the amplitude of neck muscle activity between states. EMG, electromyogram. *Indicate mean data significantly different with *P* < *0.05*. Data are presented as mean ± SEM.

Behavioural profiling identified above indicated that sedation is distinct from non-REM sleep as the animal spent more time in sedative states than it usually did in sleep, and that sedation is less fragmented than non-REM sleep. To provide a physiologically relevant assessment of sedation, we quantified the electrocortical changes induced by fentanyl by measuring the electrocortical signal. In rodents, anaesthesia is characterized by reduced locomotor activity and distinct changes in electrocortical activity^26^. We therefore compared electrocortical spectral activities associated with fentanyl sedation with those observed in wakefulness or non-REM sleep (Fig. 2). We performed spectral analyses of the electrocortical signal (Fig. 2A) and measured the electrocortical powers of 5 different frequency bands from 1 to 30 Hz (Fig. 2A,B). After control injection, rats showed an initial increased in motor activity for about 3–4 min, followed by normal behaviour and successive episodes of wakefulness and sleep similar to what was observed in previous studies in freely-behaving rats^9,17^. During non-REM sleep, δ power was higher than in wakefulness (*P* = *0.039*, n = 7, Fig. 2C), whereas β_2_ power was lower (*P* = *0.003*, n = 7). REM sleep showed lower δ activity and higher β_2_ activity than non-REM sleep (*P* = *0.081* and *P* < *0.001*, Fig. 2C). Fentanyl increased δ power (*P* < *0.001*, n = 7), decreased α (*P* = *0.012*, n = 7), and decreased β_2_ power (*P* < *0.001*, n = 7) compared to wakefulness (Fig. 2C). Compared to non-REM sleep, sedation was characterized by reduced α power (*P* = *0.047*). Sedation also differed from REM sleep as shown by higher δ (*P* < *0.001*) and θ (*P* = *0.016*) powers, and lower α (*P* = *0.006*), β_1_ power (*P* = *0.032*) and β_2_ (*P* < *0.001*) powers. In summary, fentanyl induced a sedative state that is distinct from non-REM and REM sleep, and characterized by increased δ and θ powers, and decreased α, β_1_ and β_2_ powers, when compared to wakefulness.

### Respiratory changes induced by fentanyl

**Figure 3:**
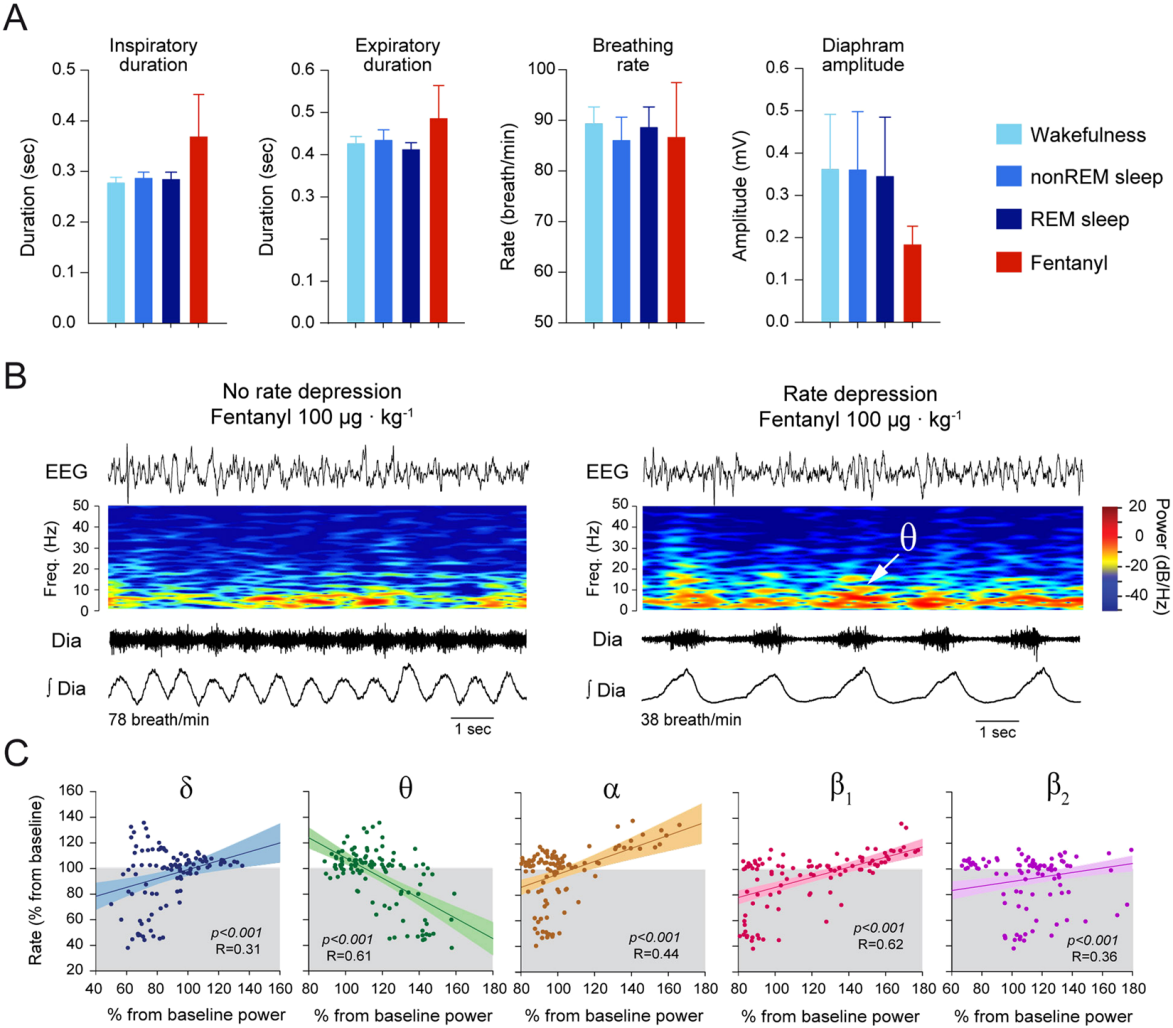
Associations between electrocortical spectral changes and respiratory rate depression induced by fentanyl. **(A)** Comparisons of inspiratory duration, expiratory duration, respiratory rate, and the amplitude of diaphragm muscle activity between wakefulness, non-REM, REM sleep, and sedation by fentanyl (100 µg · kg^−1^) did not show significant differences. (**B)** Representative epochs showing electrocortical, spectrogram and diaphragm muscle activities showed some epochs did not present respiratory rate depression whereas others did. In the latter epoch, fentanyl also increased θ power. (**C**) Correlations between the magnitude of respiratory rate depression and changes in electrocortical band powers showed that respiratory rate depression was associated with increased θ power, and decreased δ, α, β_1_, and β_2_ powers. The association between respiratory rate depression and increased θ power was highly significantly and more pronounced than for the other powers. Dia, diaphragm. Data are presented as mean ± SEM.

Here we first compared respiratory variables between wakefulness, non-REM sleep, REM sleep, and sedation. To minimize the potential effects of the initial behavioural changes induced by the intra-peritoneal injection, we looked at the mean values of the respiratory variables 30 min after injection over a duration of 15 min. Overall, fentanyl did not significantly change inspiratory duration (*P* = *0.420*, n = 7, Fig. 3A), expiratory duration (*P* = *0.649*, Fig. 3A), respiratory rate (*P* = *0.981*, Fig. 3A) compared to the values obtained in non-REM sleep and wakefulness after control injection. Although the mean diaphragm amplitude tended to be lower during sedation, it was not significantly lower than the other groups (*P* = *0.673*), because of the high variability of diaphragm amplitude in these groups. The average respiratory variables measured over 15-min periods, however, can potentially miss the transient events occurring over shorter time periods. Accordingly, we averaged respiratory and electrocortical spectral variables for each minute between 30 and 45 min following fentanyl injection, a time window when fentanyl was inducing its strongest effects. We first observed that for some 1-min periods, respiratory rate was unchanged, whereas for others respiratory rate was significantly reduced by fentanyl (Fig. 3B). We then looked at the correlations between electrocortical band power changes and respiratory rate changes. We identified significant correlations (Fig. 3C) between respiratory rate changes and δ power (*P* < *0.001*, R = 0.31, n = 112 1-min periods), θ power (*P* < *0.001*, R = 0.61, n = 112), α power (P < 0.001, R = 0.44, n = 112), β_1_ power (*P* < *0.001*, R = 0.62, n = 112), and β_2_ power (*P* < *0.001*, R = 0.36, n = 112) changes. The regressions between changes in θ or β_1_ powers and respiratory rate depression had the highest R values. In addition to a high R value, the regression for θ power showed that a two-fold increase in power was associated with a 60% reduction in respiratory rate. Therefore, although all five correlations were statistically significant, only increases in θ power were associated with significant and physiologically-relevant changes in respiratory rate (Fig. 3C).

**Figure 4:**
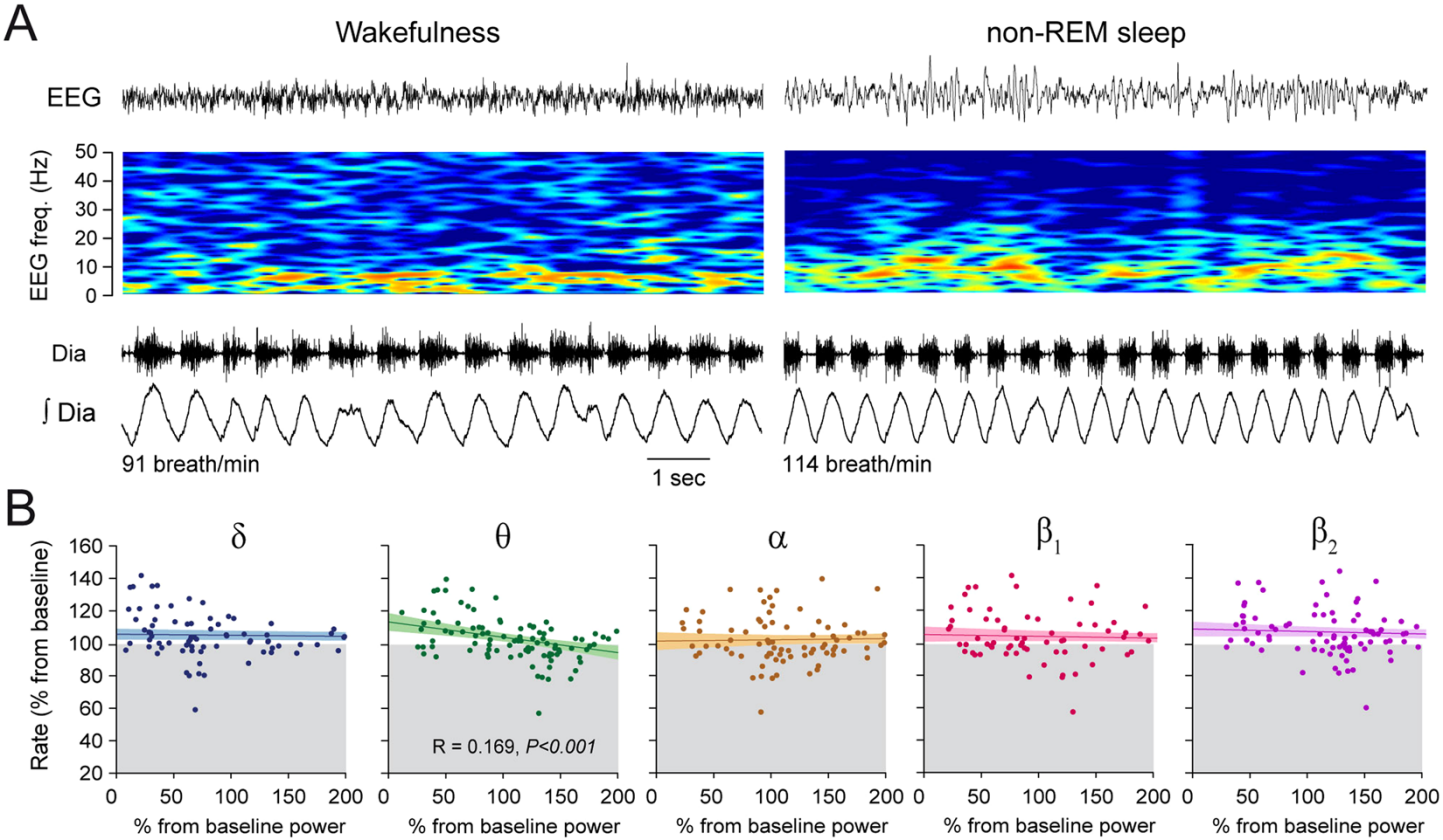
Weak associations between electrocortical changes and respiratory rate changes during wakefulness and non-REM sleep. **(A)** During wakefulness and non-REM sleep, there was no changes in respiratory rate, despite changes in electrocortical powers. **(B**) Weak correlations between respiratory rate and electrocortical changes were observed following control injection. Dia, diaphragm.

To determine whether the statistically significant relationships identified between electrocortical power changes and respiratory rate depression were due to fentanyl-induced electrocortical changes, but not to spontaneous electrocortical changes, we applied the same analysis to the control group. We calculated linear regressions between spontaneous changes in electrocortical powers and respiratory rate changes for epochs of wakefulness and non-REM sleep following the same approach as above (Fig. 4). We identified a significant, although weak, correlation between changes in θ power and respiratory rate (*P* < *0.001*, R = 0.169), but no correlations between respiratory rate and other power changes (all *P* > *0.071*, Fig. 4B). Although increased θ power was associated with small decreases in respiratory rate, the changes were minimal and rarely went below baseline respiratory rate (Fig. 4B).

### Similarities in electrocortical frequency content and diaphragm muscle activity

**Figure 5:**
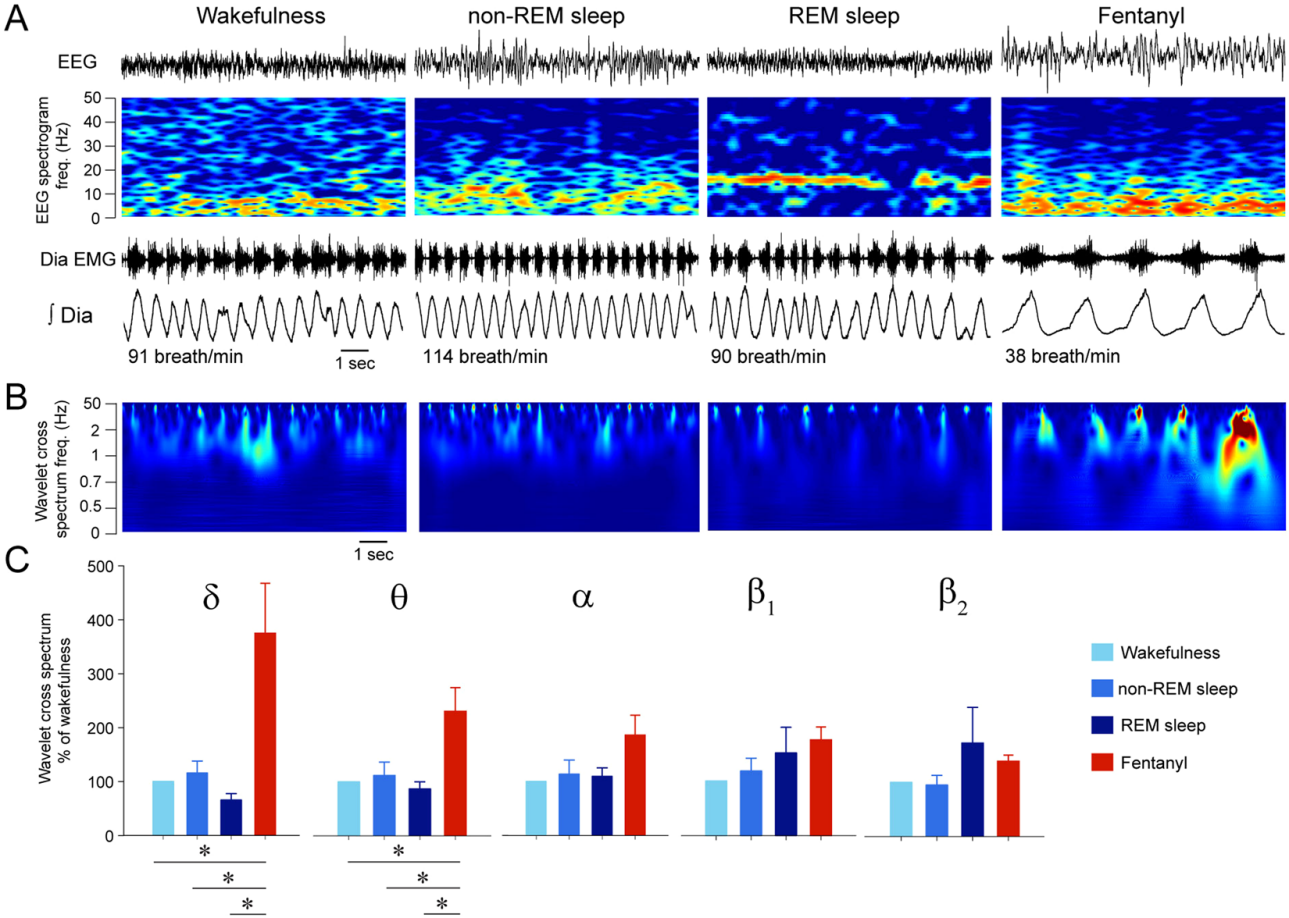
Fentanyl increases coherences between electrocortical spectral power and diaphragm muscle activity. **(A**) The coordination of electrocortical activity and diaphragm muscle activity was quantified using wavelet cross spectrum. (**B**) Wavelet cross spectrum identified frequencies at which electrocortical and diaphragm activities were strongly coordinated after fentanyl was administered. (**C**) For each power band, the cross-spectrum index was calculated and was significantly increased by fentanyl for δ and θ band powers, but not for α, β_1_ and β_2_ powers. Data are presented as mean ± S.E.M. * show means are significantly different from fentanyl with *P* < *0.05*. Dia EMG, diaphragm electromyogram.

Correlations between changes in electrocortical power and respiratory rate depression revealed cortical signatures associated with respiratory rate depression. For instance, increased θ and decreased β_1_ powers were associated with respiratory rate depression by fentanyl. To better characterize the relationship between the electrocortical and respiratory activity, we determined whether changes in electrocortical or diaphragm muscle activities were simultaneous. To this aim, we used cross spectrum wavelet analysis^22^ to identify similarities in the frequency contents of electrocortical and diaphragm muscle activities, and whether changes in electrocortical activity were reflected in respiratory activity. Although this approach did not determine whether the relationship between EEG and respiratory activity were causal, it provided finer time resolution to resolve these relationships. Wavelet cross spectrum identifies the frequency components of the electrocortical and diaphragm signals (Fig. 5A) and identifies similarities at specific frequencies. By applying this approach to electrocortical and diaphragm muscle activities, we did not observe similarities during wakefulness, non-REM sleep and REM sleep (Fig. 5B). Importantly, during fentanyl, however, there were clear changes in the cross spectrum index when the diaphragm muscle was activated, demonstrating that, as in this example, diaphragm muscle and electrocortical activity at frequencies between 0.5 and 10 Hz were synchronized. Mean data from all animals showed that the similarities observed during the fentanyl condition were high in δ (*P* = *0.003*, n = 6, Fig. 5C) and θ power bands (*P* = *0.010*) compared to wakefulness, but not in α, β_1_, and β_2_ bands (*P* ≥ *0.070*). In summary, fentanyl synchronized diaphragm muscle activity and low-frequency electrocortical activity, which is consistent with the correlations associating θ powers with respiratory rate depression.

### Anaesthesia and respiratory rate depression by fentanyl

**Figure 6:**
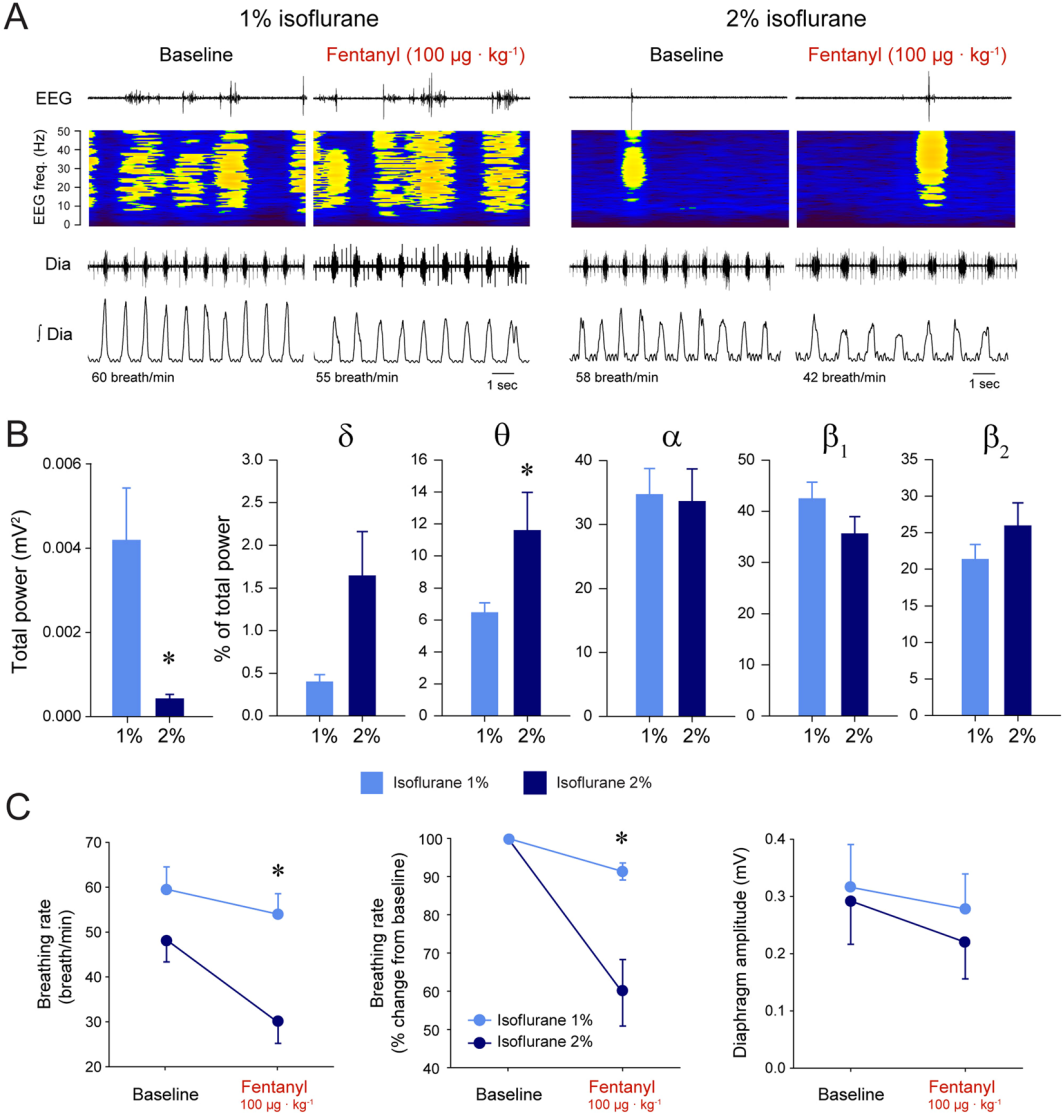
Respiratory rate depression by systemic fentanyl at different levels of anaesthesia. (**A**) Fentanyl (100 µg · kg^−1^) injection in an anesthetized rat with 1% isoflurane moderately decreased respiratory rate. The same injection of fentanyl in the presence of 2% isoflurane substantially decreased respiratory rate. (**B**) Increased isoflurane levels decreased total electrocortical power between 0–50 Hz. More detailed analysis identified that increased isoflurane decreased θ powers (n = 5), without significantly changing the other frequency bands. (**C**) Respiratory rate reduction was more pronounced at 2% of isoflurane compared to 1%. There were no significant differences between the reductions of diaphragm amplitude by fentanyl observed at the two isoflurane levels. * indicates values significantly different between isoflurane levels with P < 0.05. Data are presented as mean ± SEM.

To artificially change electrocortical activity and to determine whether it would affect the severity of respiratory rate depression, we determined the effects of fentanyl at two different levels of anaesthesia (Fig. 6A). Volatile anesthetics, such as sevoflurane, suppress electrocortical power^27^, produce bursts of electrocortical activity, and increase low frequency electrocortical activity^26,28^. The total power of electrocortical activity between 0–50 Hz was significantly lower at 2% of isoflurane than at 1% (*P* = *0.033*, n = 5). We then compared the relative distribution of electrocortical powers for the different frequency bands and their changes when isoflurane was altered from 1% to 2%. Increasing isoflurane significantly increased θ power (*P* = *0.015*, n = 5, Fig. 6B), but had no effects on the powers in other frequency bands (*P* > *0.340*). To determine whether changes in isoflurane had a significant effects on opioid-induced respiratory rate depression, we applied a repeated-measured two-way ANOVA to respiratory rate with isoflurane and opioid administration as the two factors. Respiratory rate depression was more pronounced in the presence of 2% isoflurane than at 1% (*P* = *0.036*, n = 5, Fig. 6C). With 1% isoflurane, fentanyl, at the same dose as that used in freely-behaving rats (100 µg · kg^−1^), did not significantly reduce respiratory rate (*P* = *0.250*, n = 5, Fig. 6A,C). However at 2% isoflurane, fentanyl significantly reduced respiratory rate by 42% (*P* = *0.001*). There were no significant effects on diaphragm amplitude changes (*P* = *0.082*, n = 5, Fig. 6C). In summary, these data showed that increasing anesthetic level from 1% to 2% isoflurane was associated with increased θ powers, and increased respiratory rate suppression by fentanyl.

## Discussion

The safe and optimal use of opioid analgesics is limited by the potentially life-threatening side-effects of sedation and respiratory depression associated with opioid pain therapies^29^. Because changes in arousal level associated with sleep or sedation can have significant impacts on breathing^30^, it is important to clearly identify the sedative properties of opioids and how they relate to respiratory depression. Indeed, a specific dosage of opioids may be deemed safe when the patient is awake, but its sedative effect may further aggravate respiratory depression and may lead to respiratory arrest if not treated^16,31^. In the present study, we aimed to identify the sedative properties of fentanyl by measuring behavioural and electrocortical changes in rats. Fentanyl reduced the time spent behaviorally active and induced less fragmented periods of rest, indicative of sedation. Fentanyl also induced electrocortical changes that differed from those observed in sleep or wakefulness. Fentanyl increased δ and θ powers, but reduced α, β_1_ and β_2_ powers, when compared to wakefulness. Interestingly, potent respiratory rate depression by fentanyl was only observed when θ power was increased by fentanyl. Similar to opioid sedation, isoflurane anaesthesia increased θ power and accentuated the magnitude of respiratory rate depression by fentanyl. Overall, these data showed that fentanyl-induced changes in electrocortical activity, especially in the θ power band, is tightly associated with the magnitude of respiratory rate depression.

### Behavioral and electrocortical properties of sedation by fentanyl

In patients, sedation is classically assessed using medical scoring systems^32^ and/or responsiveness to external stimulus^33^. Although objective measurement systems, such as electroencephalography, could better monitor sedation than scoring systems, these systems are not validated for clinical use and interpretation of the EEG is problematic^32^. In animals, sedation can be assessed using the loss of righting reflex which requires animal manipulation^5,34^ which is not feasible in the context of respiratory measurements in freely-behaving rodents. Here, using a combination of behavioural/motor assessments and electrocortical recordings, we identified the sedative properties of fentanyl. Our behavioural data showed that fentanyl reduced behavioural activity, decreased sleep-wake state fragmentation, the number of brief electrocortical arousals, and eliminated REM sleep, consistent with sedation and with previous studies^35,36^. In addition, the relatively high dose of fentanyl used in this study increased δ electrocortical power, but reduced α and β_2_ powers. Importantly, these electrocortical changes observed with opioid sedation differed from the changes induced by sleep, where as established, δ power increases and β power decreases during non-REM sleep, whereas θ power increases during REM sleep^25^. Consistent with our data, fentanyl administered to rats at similar dosage (150 µg · kg^−1^ increased low frequency electrocortical power^37^. In humans, sulfentanyl, a potent µ-opioid receptor agonist, induces dose-dependent changes in EEG activity. At a mild dose, the EEG shows reduced high-frequency β power and increased α activity^15^. With increasing doses of sulfentanyl, EEG increased θ and δ EEG powers. Similarly to sulfentanyl, fentanyl administered to human subjects decreased high frequency EEG activity^16^ and increased θ power^38^, which is consistent with our data in rats. It cannot be excluded that arousal levels and electrocortical activity may be altered by increased arterial pCO_2_ due to the respiratory depression induced by 100 µg · kg^−1^ of fentanyl^21^. However, increased CO_2_ levels have been shown to de-synchronized EEG activity^39^, which is not consistent with our data showing increased synchronized θ and δ powers.

### Mechanisms of sedation

Activation of µ-opioid receptors by opioids produces potent analgesia because of their main effects on nociceptive pathways. Opioids inhibit ascending nociceptive circuits^40^ and enhance descending inhibition of nociceptive inputs^41^. The descending nociceptive pathway, which comprises the midbrain periaqueductal gray, and its descending projections to the rostral ventromedial medulla and spinal cord, forms an essential neural circuit for both endogenous and exogenous opioid-mediated analgesia^42^. By binding to µ-opioid receptors in the periaqueductal gray^42^ and/or the rostro-ventral medulla^41^, opioids disinhibits GABAergic circuits, which leads to reduced nociception^43^. Importantly, nociceptive pathways also modulate arousal and their inhibition by opioids induces sedation^14^. In fact, inhibition of periaqueductal gray GABAergic neurons promotes non-REM sleep and produces slow-wave electrocortical activity^44^. The opioid-induced δ and θ electrocortical power increases observed in the current study may therefore be due to inhibition of periaqueductal gray neurons by opioids. Alternatively, opioids also decrease arousal by inhibiting brainstem cholinergic circuits^45^ at the level of the lateral dorsal tegmental nucleus, the pedunculopontine tegmental nucleus, the medial pontine reticular formation, and thalamus^46^. The opioid-induced reductions of α and β powers observed in the present study are consistent with inhibition of pontine cholinergic neurons by fentanyl.

### Associations between electrocortical changes and respiratory depression

Fentanyl-induced sedation is associated with distinct behavioural and electrocortical changes. In addition to characterizing the sedative state induced by fentanyl, these electrocortical signatures can also be linked to respiratory changes^16^. Using correlations between electrocortical changes and the magnitude of respiratory rate depression, we found that increased θ powers and decreased β_1_ powers were associated with respiratory rate depression, and these relationships were significantly weaker in other electrocortical frequency bands, despite the fact that δ, α, and β_2_ powers were also all altered by fentanyl. In other words, only when θ power was increased, respiratory rate was substantially depressed by fentanyl. This association was not observed when θ power naturally changed across sleep-wake states. This relationship suggested that it was the fentanyl-induced θ changes that had significant effects on breathing. A key-circuit modulating nociceptive pathways and opioid analgesia is the reticular formation^40^. The reticular formation comprises the periaqueductal gray and the rostral ventromedial medulla, two structures involved in arousal^14^ and the regulation of breathing^7,47^. The periaqueductal gray regulates pain^41^, it expresses μ-opioid receptors, and is inhibited by fentanyl^48^. It also presents reciprocal connections^49^ with the pre-Bötzinger complex, a small population of cells in the medulla contributing to opioid-induced respiratory rate depression^9^ and critical for breathing generation^50^. The reticular formation, including the periaqueductal gray, provides excitatory inputs to respiratory neurons during wakefulness^51,52^ and its inhibition by opioids may reduce its impacts on respiratory neurons. Under those inhibitory conditions, the respiratory network is only vulnerable to local brainstem inhibition, which may explain why respiratory rate depression was only observed in states of reduced brain arousal^9^. Finally, the periaqueductal gray may be a key component mediating analgesia, reduced arousal, and respiratory depression by opioids, and may constitute a link between electrocortical changes and respiratory depression observed in the present study.

### Importance of arousal states and behaviours when assessing respiratory depression

The severity of respiratory depression by opioid analgesics and the identification of potential new therapies with limited respiratory side-effects using pre-clinical models depend on the quality of respiratory assessments as well as a clear characterization of their sedative properties. In a recent study, respiratory depression by novel analgesics was quantified in freely-behaving rodents without simultaneously assessing sedation^20^. These results proved to be misleading because a recent study using adequate controls to quantify respiratory depression showed that PZM21 presents respiratory depressant effects compared to other opioids^53^. Although it is unknow whether the respiratory depressant effects of PZM21 were due to its sedative properties, it raises significant concerns on studies that only assess breathing without considering the behavioral and cortical changes associated with opioids, especially because respiratory depression may only be present when distinct electrocortical changes occur.

### Summary

In addition to their analgesic properties, opioid drugs induce sedation and respiratory depression. Here, we aimed to reveal the link between the sedative properties and the respiratory depressant effects of opioids. We first characterized the behavioral and electrocortical changes associated with sedation by fentanyl. Fentanyl reduced motor activity and altered specific electrocortical power bands that differed from the changes associated with other states of reduced arousal such as non-REM and REM sleep. Importantly, the magnitude of respiratory rate depression by fentanyl was substantially associated with increased θ electrocortical power, but only weakly with changes in other power bands. Overall, these data provide new evidence linking electrocortical changes to the severity of respiratory depression by opioid analgesics. This study also highlights the importance of considering the cortical and subcortical effects of opioids in addition to their impacts on breathing when evaluating opioid-induced respiratory depression. Our data provide clinically relevant evidence of the impact of fentanyl on arousal and breathing. The dose of fentanyl used here (100 µg/kg) is equivalent to a relatively high dose of fentanyl in humans (4–6 µg/kg)^21^ and induces severe sedation with respiratory rate depression only happening when distinct cortical changes occurred.
